# Classifying avian drinking behaviour: ecological insights and implications in a changing world

**DOI:** 10.1002/brv.70150

**Published:** 2026-02-22

**Authors:** Shannon R. Conradie, Marc T. Freeman

**Affiliations:** ^1^ School of Animal, Plant, and Environmental Sciences, University of the Witwatersrand 1 Jan Smuts Ave, Braamfontein Johannesburg 2001 South Africa; ^2^ Department of Zoology and Entomology University of Pretoria Lynnwood Rd Pretoria 0002 South Africa; ^3^ South African Research Chair in Conservation Physiology, South African National Biodiversity Institute 2 Cussonia Ave, Brummeria Pretoria 0184 South Africa

**Keywords:** drinking, thermoregulation, avifauna, behavioural ecology, eco‐physiology

## Abstract

Water is a fundamental currency of life, and its availability significantly influences animal behaviour, physiology and distributions. However, our knowledge around the dependence on water for drinking and the direct and indirect mechanisms driving related behaviours remains partial in the context of changing climates. Here, we review patterns and classifications of avian reliance on water consumption and the role of abiotic and biotic factors in drinking habits. We bring to light the void of information regarding drinking habits in relation to thermal physiology, and then review existing literature related to the behaviour of drinking. We attempt to highlight misconceptions related to the typical classification of drinkers *versus* non‐drinkers and direct future research efforts to improve our understanding of the dynamic relationship between water availability, acquisition, and maintenance under climate change. We conclude that despite the common dichotomous use of drinking *versus* non‐drinking classifications in climate vulnerability assessments, this trait is highly flexible and occurs along a continuum from specialist non‐drinking species to highly dependent drinking species driven by abiotic and biotic pressures. While some recent assessments continue to apply binary traits, our gradient‐based approach advances ecological relevance by accounting for context‐dependent flexibility. Consequently, previous approaches assessing drinking *versus* non‐drinking species may not be as ecologically relevant as previously thought. Improving our understanding of the spatial and temporal trends in how species acquire or use water for drinking, along with sound empirical data, will continue to shape our theoretical understanding of this trait. We emphasise that understanding drinking as a trait is critically important for comprehending species vulnerabilities to anticipated future climates, especially given predicted changes and increased variability in water availability.

## I. INTRODUCTION

*It is doubtful that any bird preferentially refrains from drinking and utilises a dry diet if water or succulent food is available* (Dawson, 1982, p. 498).

When, why, and how an animal consumes water is complex, idiosyncratic and often misunderstood. While it is generally accepted that water plays a fundamentally important role in shaping animal behaviour and survival (Davies, 1982; Lopez *et al*., 2023), the relationship between water consumption and thermoregulation, particularly during extreme heat, is poorly understood. For many endotherms, water acquisition – particularly in liquid form – is crucial for facilitating thermoregulation through evaporative water loss (EWL), when environmental temperatures exceed body temperature (Lasiewski, Acosta & Bernstein, 1966; Studier, 1970; Dawson, 1982). Consequently, survival during exposure to extreme air temperatures (*T*_air_) hinges on the maintenance of both energy and water balance to avoid lethal hyperthermia and/or dehydration (Riddell *et al*., 2019). In recent decades, endotherms have increasingly been reported to succumb to heat‐related mass‐mortality events driven by these mismatches between energy, water supply and demand (Welbergen *et al*., 2008; Ratnayake *et al*., 2019; McKechnie *et al*., 2021*b*). Replenishing water reserves lost *via* EWL during sustained hot weather is fundamentally important, but the ability to do so is constrained by abiotic (e.g. water availability, thermal landscape) and biotic factors (e.g. evaporative cooling efficiency, behavioural thermoregulation) (Rozen‐Rechels *et al*., 2019; Boyle, Shogren & Brawn, 2020; Juillard *et al*., 2025). Over 70 years of long‐term monitoring data now reveal that the intensification of extreme heat events, escalating approximately tenfold at low latitudes over recent decades, has already caused 25–38% reductions in tropical bird abundance through hyperthermia and dehydration, highlighting the pressing need to refine our understanding of water‐dependent traits for conservation (Kotz, Amano & Watson, 2025). Moreover, understanding the relationship between water acquisition, allocation and maintenance is critically important for comprehending how birds operate within heterogeneous thermal landscapes (Bartholomew & Cade, 1963), but is complicated by animal behaviour and physiology.

Consumption of water varies (often unpredictably) between and even within species (McCluney, 2017), ranging from those that are highly dependent on drinking to those that seem not to drink at all (Fisher, Lindgren & Dawson, 1972; Lynn, Rosenstock & Chambers, 2008; Smit *et al*., 2019). Dependence on water – typically dichotomised into drinking *versus* non‐drinking (e.g. Czenze *et al*., 2020) – could be an informative and valuable trait that sheds light on the vulnerabilities of assemblages as they navigate a climatically unstable future. Nevertheless, the reliability and subjectivity involved in classifying species as drinkers *versus* non‐drinkers remains unclear, raising questions about the feasibility of these classifications and their potential contributions to our understanding of ecological phenomena and use in climate vulnerability assessments.

Our understanding of avian drinking habits remains limited and inconsistent, and the research on this topic to date has mostly focused on arid and semi‐arid systems where surface water is scarce (Fisher *et al*., 1972; Abdu, Lee & Cunningham, 2018; Czenze *et al*., 2020; Orolowitz, Shadwell & Cunningham, 2023). Consequently, we know far less about drinking behaviour and water dependency in mesic or tropical environments where water is generally more readily available (but see Delgado‐Martínez *et al*., 2023; Skead, 1975*a*). Rising humidity in tropical lowlands is projected to intensify lethal hyperthermia risks by limiting evaporative cooling scope, even for resting birds, potentially increasing hyperthermia risk five‐ to tenfold by 2050 and posing a serious threat to global avian diversity (Coulson *et al*., 2025). These limitations on evaporative cooling will likely increase drinking demands as birds attempt to maintain water balance.

Here, we aim to review and discuss our current knowledge on the drinking of water among birds and place it into a behavioural, physiological, and spatio‐temporal context. We firstly attempt to address the biological role of drinking and evaluate critically whether the typical dichotomous classification of drinking *versus* non‐drinking is ecologically relevant, or if this relationship is better understood as a continuum driven by abiotic and biotic pressures. Secondly, we explore whether drinking can be used as a reliable trait to assess species vulnerabilities to changing climates and landscapes. Finally, we discuss emerging questions and future research opportunities to investigate what determines drinking behaviour and what the ecological relevance of this is in a rapidly changing world.

## II. DRINKING AND THERMOREGULATION

Despite significant advancements in our understanding of avian thermal physiology, critical gaps remain in our knowledge around the role of drinking behaviour/water acquisition and its subsequent effects on patterns of avian thermoregulation. Bartholomew & Cade (1963) previously emphasised a surprising lack of quantitative and experimental research focused on aspects of water use related to avian biology prior to the 1950s, which still resonates today. While the complexities around the mechanisms of water acquisition and loss are generally well understood (Bentley, 1970; Takei, 2015), investigations into how variation in drinking behaviours (i.e. drinking *versus* non‐drinking birds) affects traits of avian thermoregulation (e.g. body temperature maintenance, evaporative water loss, metabolic rate) remain scarce (but see Czenze *et al*., 2020; J.C. Short, M.T. Freeman, S.J. Cunningham, A.E. McKechnie, unpublished data). Physiologically, both intra‐ and inter‐specific variation in drinking behaviour may be of paramount importance, particularly for species occupying regions where projected climate change is likely to impose serious constraints on their long‐term survival.

Regardless of habitat type or climatic zone, survival typically depends on maintaining a positive water balance; a requirement that is particularly critical for effective thermoregulation (Lasiewski *et al*., 1966; Dawson, 1982). Maintaining a balanced water economy can be challenging, particularly in arid environments where water and food availability are scarce, unpredictable and costly (Dawson & Schmidt‐Nielsen, 1964). To use water effectively for thermoregulation, birds must have access to adequate water reserves. Birds use three primary sources of water: free‐standing water, preformed water found in food, and/or metabolically produced water (Robbins, 1993; Smit *et al*., 2019). Free‐standing water refers to any liquid water that birds may drink or encounter directly, including ephemeral pools, seasonal pans, streams, wetlands, springs, dew, and anthropogenic sources such as troughs or dams (Rozen‐Rechels *et al*., 2019). The reliance on preformed water is shaped by season and diet (Bartholomew & Cade, 1963; Macmillen, 1990). For instance, the amount of water derived from green grass shoots, sprouting seeds, and insects during the wet season contrasts with a dry seed‐based diet typical of the dry season (Willoughby & Cade, 1967). The role of season and diet on drinking dependence is reviewed in detail below (Section IV; Table 1). Alternatively, some birds may lack access to drinking water, either permanently or temporarily (see Sections IV.1 and IV.2), and may resort largely to metabolic water production (i.e. a by‐product of metabolic processes, primarily during the oxidation of carbohydrates and fats) (Dawson, 1982). Birds have a remarkable capacity for water production, largely due to their high metabolic rates, and for water conservation through the excretion of nitrogenous waste as uric acid rather than urea. Uric acid is both less toxic and soluble in water and can be excreted in a semi‐solid form, thus conserving water (Lee & Schmidt‐Nielsen, 1971; Fisher *et al*., 1972; Laverty & Skadhauge, 2008). Such adaptations are particularly beneficial in arid environments where water availability is limited and allow birds to maintain a water economy that enables them to persist across a diverse array of habitats, from deserts to rainforests (Barsoum & El‐Khatib, 2017).

**Table 1:** Typical water content of diet per dietary guild and the potential implications for drinking dependence in birds. These implications likely vary within categories and are not discrete or fixed but exist along a continuum.

Dietary guild	Typical water content (%)	Examples	Potential implications for drinking dependence
Granivorous (dry seeds)	~10	Finches, larks	High; often regular or obligatory drinkers due to low water in diet (Skead, 1975*b,a*).
Insectivorous	50–85	Warblers, flycatchers	Low; typically non‐drinkers or opportunistic and may vary seasonally (Bartholomew & Cade, 1963).
Frugivorous	70–90	Bulbuls, mistletoebirds	Low to moderate; seasonal shifts possible (Prozesky, 1975).
Carnivorous/high‐protein	50–85 (variable with prey)	Eagles, shrikes	Moderate; can increase to high if protein elevates uric acid demands (Cade, 1965; Fisher *et al*., 1972)
Nectarivorous	80–95	Hummingbirds, sunbirds	Very low; nectar supplies most hydration (Nicolson, 2002).

During bouts of intense activity or when *T*_air_ > body temperature (*T*_b_), birds will dissipate heat through EWL, a process that cools the body through the loss of water vapour to maintain *T*_b_ below lethal limits (Dawson, 1982; McKechnie & Wolf, 2019). Unlike their mammalian counterparts, birds lack sweat glands (i.e. eccrine glands) and therefore rely predominantly on respiratory evaporative water loss through mechanisms such as panting or gular flutter (Bartholomew & Lasiewski, 1968; Czenze *et al*., 2021; McKechnie, Gerson & Wolf, 2021*a*), and/or cutaneous evaporative water loss for thermoregulation (Bernstein, 1971; Lasiewski, Bernstein & Ohmart, 1971; Ohmart & Lasiewski, 1971; McKechnie *et al*., 2016). However, when birds have limited or no opportunities to drink, they likely experience strong trade‐offs between thermoregulation, hydration state and activity levels with increases in *T*_air_ (Speakman & Król, 2010; Smit & McKechnie, 2015). For instance, among arid‐zone larks, the absence of drinking water favours species with lowered metabolic heat production, which aids in conserving water expenditure for evaporative cooling (Tieleman, Williams & Visser, 2004).

The evolutionary drivers underlying why some bird species regularly consume surface water while others do not, or do so only inconsistently, remain poorly understood. Some studies suggest that smaller birds tend to exhibit higher relative water consumption (Cooper *et al*., 2019), which is attributed to their greater surface area to volume ratios that result in increased rates of EWL (Woods & Smith, 2010). For instance, hummingbirds (Trochilidae), with their small size and high metabolic demands, must frequently consume nectar to satisfy both their energy and hydration needs (Collins & Paton, 1989). By contrast, larger birds, possessing a smaller surface area to volume ratio, may have lower relative water requirements but still depend on reliable access to water sources to prevent dehydration, particularly when exposed to acute or chronic periods of extreme heat (Whitfield *et al*., 2015).

To assess the influence of drinking behaviour on avian thermal physiology, it is necessary to categorise species according to their drinking habits. Reliable classification is essential to ensure inferences regarding avian water economy, thermal physiology and associated ecological dynamics, including vulnerabilities to shifting water availability and climatic change, are informative and valid. Misclassifying birds as drinkers or non‐drinkers could have significant implications for our broader understanding of avifaunal vulnerabilities that may arise if access to water or dietary sources for these species changes (Boyle *et al*., 2020). For instance, Czenze *et al*. (2020) found that the ratio of maximum EWL to minimum thermoneutral EWL (i.e. evaporative scope) during acute heat exposure among 12 African passerines was significantly higher for drinking species compared to non‐drinking species. The authors subsequently showed that raised evaporative scope among drinking species is functionally linked with raised heat tolerance limits and larger increases in total EWL than in non‐drinking species (Czenze *et al*., 2020). Such findings are valuable and provide novel insights into the thermoregulatory capacity of arid‐zone species. However, studies such as these that explicitly contrast and examine the differences between drinking and non‐drinking birds in terms of thermal physiology remain limited, particularly in non‐arid environments.

The classification of birds as drinkers *versus* non‐drinkers, and its implications for thermoregulation, adds an important layer of complexity to our understanding of avian vulnerabilities to climate change. However, the criteria for categorising these behaviours are not well established, highlighting the need for further research into the environmental and physiological factors that influence drinking behaviour across various avian taxa. Moreover, the impacts of climate change and habitat degradation pose additional questions about water availability and consumption patterns among birds. As specific habitats become drier or increasingly impacted by human activities (IPCC, 2021), the strategies birds employ for water acquisition may evolve, underscoring the necessity for longitudinal studies that capture behavioural changes over time. A deeper understanding of these behaviours, and their influence on avian thermal physiology, is crucial both for advancing fundamental knowledge and for informing conservation strategies in the face of an uncertain ecological future.

## III. BEHAVIOURAL DETERMINANTS OF DRINKING

**Fig. 1:**
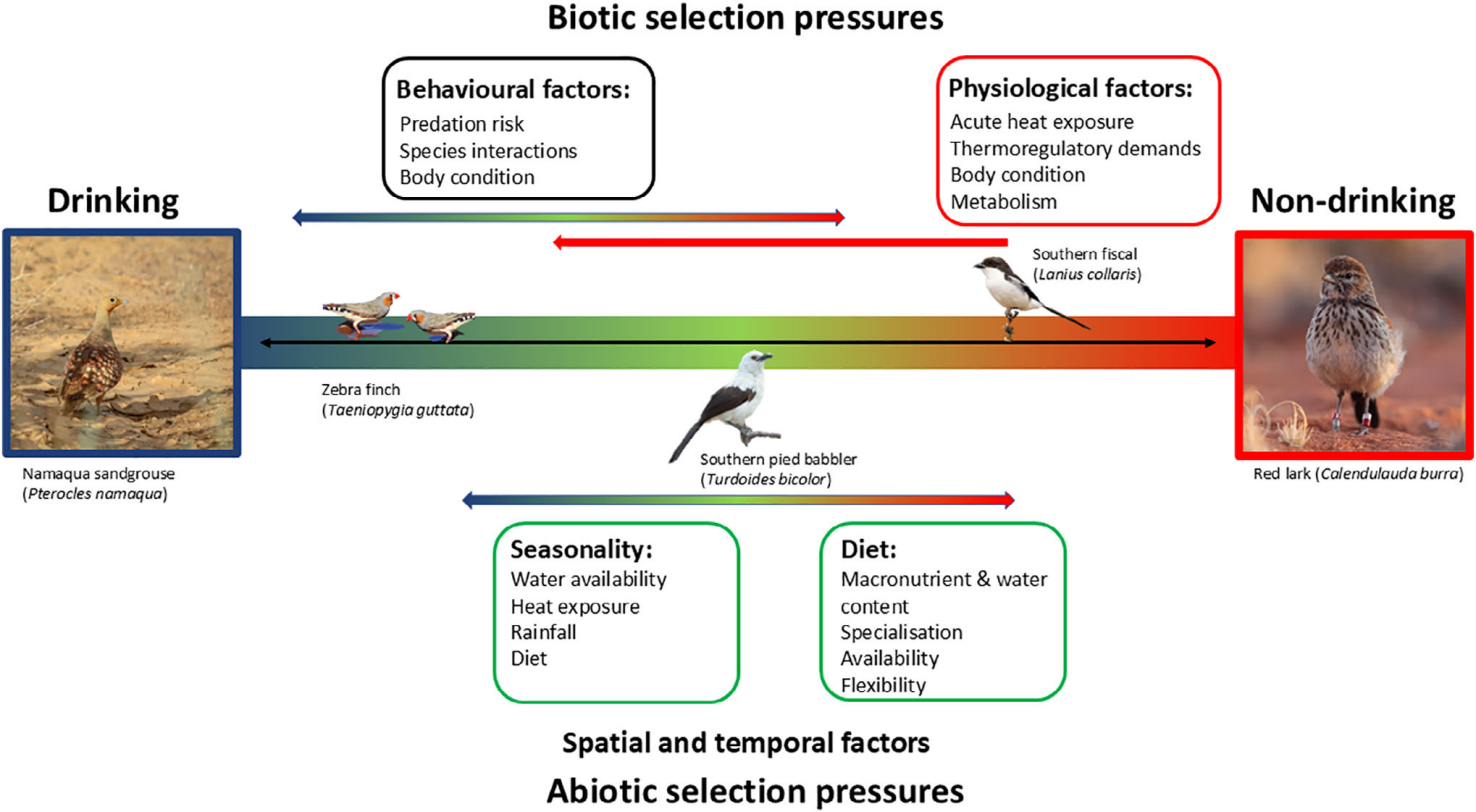
Drinking habits classification in relation to thermal physiology, behaviour and the environment. The classification schematic illustrates drinking *versus* non‐drinking birds as a continuum with abiotic (i.e. seasonality and diet) and biotic (i.e. behaviour and physiology) pressures driving species placement between one end and the other rather than being a dichotomous classification. Bird photographs courtesy of: W. Tarboton; L. Harris and M.T. Freeman.

Avian behaviour is shaped by several factors, notably the need to maintain energy and water balance in response to fluctuating environmental conditions (Sears *et al*., 2016; Albright *et al*., 2017). Under extremely hot conditions, birds manage their heat loads by modifying behaviour (i.e. behavioural thermoregulation) to reduce thermoregulatory costs experienced when energy and water requirements are exceeded (e.g. dehydration or hyperthermia). Drinking also provides a mechanism for birds to avoid mismatches between water supply and demand. However, understanding this relationship and the factors influencing drinking is complicated by abiotic (e.g. water availability, thermal landscape) and biotic factors (e.g. thermoregulatory demand; Fig. 1).

The reliance on drinking water is defined by a species' ecology, which may influence patterns of thermoregulatory behaviour and how birds use the thermal landscape (Fisher *et al*., 1972; Orolowitz *et al*., 2023). For instance, in the absence of drinking, birds may seek cooler microsites to reduce heat exposure, whereas individuals that regularly drink may tolerate riskier thermal behaviour (e.g. foraging in direct solar radiation) that would otherwise be unsustainable under hot conditions (Orolowitz *et al*., 2023; A.R. Bourne, personal communication, 2019). Moreover, Orolowitz *et al*. (2023) demonstrated that regularly drinking birds in the Tankwa National Park of South Africa engage in panting at lower *T*_air_ than their non‐drinking counterparts. Behavioural differences between drinking and non‐drinking species may also be linked to fundamental differences in their physiology (see Section II; e.g. Czenze *et al*., 2020).

Whether an individual drinks or not may also vary depending on sex and/or breeding status. For instance, some birds (e.g. hornbills) enclose females inside the nest cavity during incubation and early chick development, without direct access to water, making them entirely reliant on the dietary items provisioned by the male (Kemp, 1995). Despite such clear potential for sex‐based differences in drinking ecology, this aspect of avian water use remains underreported and largely unquantified. Drinking behaviour may also vary across age groups. For example, most altricial nestlings typically obtain water from provisioned food and do not drink water directly (but see Lahti & Barlow, 2024), while the adults of these species may drink (Dunn, 1975; Olson, 1992). By contrast, precocial species may exhibit early independence in water acquisition, with parents facilitating access to free‐standing water shortly after hatching to support rapid growth amid environmental stressors (e.g. heat exposure). In some exceptional cases, such as in sandgrouse (e.g. Namaqua sandgrouse, *Pterocles namaqua*), males frequently transport water in specially modified breast feathers over long distances to their nestlings for consumption (Cade & Maclean, 1967).

As global *T*_air_ increases, the reliance on water for species that regularly drink may increase, while those not dependent on water may be forced to drink under conditions where evaporative cooling demands exceed metabolic water available for thermoregulation. For instance, small insectivores such as grey tit‐flycatchers (*Myioparus plumbeus*) and willow warblers (*Phylloscopus trochilus*) are typically categorised as non‐drinkers but have been reported to drink on extremely hot days in northern Kwa‐Zulu Natal, South Africa (A.S. Riley, personal communication, 2019). Similarly, southern fiscals (*Lanius collaris*) were only reported to drink water on days where daily maximum *T*_air_ >35 °C (Smit, 2013). Increased time spent drinking around water holes may expose birds to additional thermoregulatory and predatory vulnerabilities. Indeed, several mass‐mortality events have been documented to occur around water holes (Finlayson, 1932; Towie, 2009) likely as a consequence of elevated hyperthermia risk arising from the combination of activity and high solar heat loads, and indirect radiation reflected from water surfaces.

Free‐standing water bodies are also associated with higher levels of predation, particularly in arid environments (Fisher *et al*., 1972; Ferns & Hinsley, 1995). In these systems, aerial predators (e.g. goshawks, sparrowhawks, falcons, etc.) commonly perch near waterholes to ambush species that are reliant on drinking free‐standing water (Cade, 1965), while ground predators (e.g. snakes, mongoose) frequently wait or conceal themselves near free‐standing water to capture those visiting (Lazarus & Symonds, 1992). Species facing substantial predation risk, and that are highly reliant on drinking, often exhibit specific behavioural adaptations to reduce their vulnerability during drinking activities (Fisher *et al*., 1972). For instance, social species will congregate around water sources to increase vigilance and reduce individual predation risk (Roberts, 1996). However, not all species regularly visiting water sources face substantial predation risk. One such exception is drinking birds from the fynbos biome of the Western and Eastern Cape of South Africa, which experience low levels of predation risk near water sources despite daily visits (Lee, Wright & Barnard, 2017).

Shifts in visitation rates to water sources may also expose species to novel interspecific interactions. These interactions may increase competition and, by extension, negatively impact water balance for less‐aggressive species. For example, nectarivores are typically more aggressive in their foraging behaviour and, if this behaviour is upheld for drinking, these species may constrain the ability of small, highly water‐dependent species to maintain water requirements (Mac Nally & Timewell, 2005). By contrast, increased interspecific interactions at water sources may increase vigilance and reduce the probability of being predated.

Some drinking species may also be able to withstand long periods without drinking by shifting their diets to feed on water‐rich resources. For instance, inland dotterel (*Peltohyas australis*) often feed on succulent plants during the day, where they replenish body water and reduce the necessity to drink when water is scarce (Davies, 1982). By contrast, granivores who consume a predominantly dry diet are typically classified as highly reliant on drinking (Fisher *et al*., 1972; Williams & Koenig, 1980), resulting in selection of habitat near water sources. For instance, Kotler, Dickman & Brown (1998) showed that when water was available, Australian ravens, *Corvus coronoides*, being fed peanuts were less likely to abandon foraging efforts. The influence of diet on drinking behaviour, and the temporal and seasonal drivers of dietary water acquisition, are reviewed in Section IV.

In addition to direct water consumption (i.e. drinking), birds may be dependent on water sources for bathing or behavioural thermoregulation. For instance, birds frequently stand in shallow water during heat waves to downregulate body temperature *via* conductive heat loss (Slessers, 1970; Jullien & Thiollay, 1998; Oswald *et al*., 2008). While some of these species that engage in bathing for behavioural thermoregulation may be classified as non‐drinkers, they can still be heavily reliant on surface water for effective heat dissipation during acute heat exposure. Similarly, Jirinec (2024) demonstrated that some lowland Amazonian birds experience enhanced cooling during the wet season. For example, spotted antpitta (*Hylopezus macularius*) experienced significant cooling as rainfall increased, however, this pattern was not consistent across the species investigated (Jirinec, 2024). In particular, *Sclerurus* species experienced no correlation between cooling and rainfall, but these species typically shelter underground, allowing them potentially to avoid rainfall altogether. Birds may opportunistically use rainfall or free‐standing water for thermoregulation without necessarily consuming the water, and therefore may not be classified as obligatory, regular, or even opportunistic drinkers (Table 2).

**Table 2:** Conceptual framework for classifying avian drinking behaviour along a gradient. Categories are based on abiotic (e.g. seasonality, temperature, diet) and biotic (e.g. behaviour, physiology) influences. These classifications are not discrete or fixed but exist along a continuum from non‐drinking to obligate drinking, with species potentially falling into more than one category depending on environmental and physiological conditions.

Type of drinker	Description	Examples	References
Non‐drinkers	Species that do not drink, even when water is available. Typically obtain all their water from water‐rich dietary items (e.g. insectivores, omnivores)	Red lark (*Calendulauda burra*) Mistletoebird (*Dicaeum hirundinaceum*)	Kemp & McKechnie (2019); Fisher *et al*. (1972)
Heat‐induced drinkers	Typically do not drink, but when evaporative cooling demands exceed available water reserves for thermoregulation under hot conditions, these species will likely drink	Southern fiscals (*Lanius collaris*) Grey tit‐flycatcher (*Myioparus plumbeus*)	Votto *et al*. (2020)
Opportunistic drinkers	These species occasionally drink when water is available and may do so even on cooler days	Southern pied babblers (*Turdoides bicolor*) Scaly‐feathered weaver (*Sporopipes squamifrons*) Zebra finch (*Taeniopygia guttata*)	Smit *et al*. (2019); Prior *et al*. (2013)
Seasonal drinkers	During the dry season, these species will engage in drinking, but during the wet season, they can obtain sufficient water through their diet (often insectivores)	Cape robin chat (*Cossypha capensis*) Red‐eyed bulbul (*Pycnonotus nigricans*) Stark's lark (*Spizocorys starki*)	Prozesky (1975); Willoughby (1971)
Regular drinkers	These species regularly drink but are not completely dependent on drinking. They typically feed on dry seed and need to supplement water acquisition with surface water	Zebra finch (*Taeniopygia guttata*)	Cooper *et al*. (2019)
Obligatory drinkers	These species are fully reliant on drinking and engage in drinking daily	Sandgrouse (Pteroclidae) Lovebirds (*Agapornis*)	Ward (1972); Warburton & Perrin (2005)

The relationship between drinking and behaviour is fundamentally complicated. While changes in environmental conditions may force species to drink (Fig. 1), this may lead to novel threats and limitations on how species operate within the thermal landscape. Untangling these patterns and dependencies also depends on our understanding of the role that spatial and temporal factors have on drinking.

## IV. SPATIAL AND TEMPORAL FACTORS SHAPING DRINKING DEPENDENCE

While drinking is generally defined by a species' ecology, the extent to which a species is reliant on drinking may be shaped by abiotic (e.g. heat exposure, water availability) and biotic (e.g. thermal physiology, water economy; Fig. 1) factors. Understanding the relationship between these factors and water economies is essential for predicting how birds interact within their environments and how projected climate change will likely influence the ability of birds to maintain water balance (e.g. Conradie *et al*., 2024).

### (1) Spatial patterns of drinking

When water is limited, drinking birds disperse in relation to water availability (Tieleman *et al*., 2004) and thus the spatial arrangement of water sources shapes birds' movements and habitat use. When limited water availability is coupled with extreme heat, species such as large‐billed lark (*Galerida magnirostris*) or Dunn's lark (*Eremalauda dunni*) will favour sites with access to water over sites with high nutritional return but no water availability (Tieleman *et al*., 2004; Orolowitz *et al*., 2023).

Birds that rely on drinking have evolved strategies to use water sources effectively, reflecting their habitat use. For instance, birds that are typically ground‐foragers also exploit natural surface water such as rivers and pools, while arboreal feeders may have evolved to exploit arboreal water in tree holes or crevices (Kirsch *et al*., 2021; Delgado‐Martínez *et al*., 2023). These water‐filled tree crevices are crucial during hot summer months in temperate forests with species such as treecreepers (*Certhia* spp.), Eurasian bullfinch (*Pyrrhula pyrrhula*) and European robin (*Erithacus rubecula*) frequently visiting them for feeding and drinking (Kirsch *et al*., 2021).

Topography and elevation also influence water availability and the selection of drinking sites. In high‐elevation areas, water sources may be more abundant and consistent due to greater precipitation and cooler temperatures than lower‐elevation areas with higher temperatures and greater evaporation (Zhang *et al*., 2022). Additionally, topographical features around water sources may influence drinking. For instance, birds such as sandgrouses, which are highly reliant on water and spend large portions of their days around water sources, have been reported to avoid water holes with large areas of exposed ground, which would make them more visible to predators (Ferns & Hinsley, 1995).

Nesting success may also be influenced by the proximity of the nest site to water. For example, snow geese (*Chen caerulescens*) have reduced nesting success when evaporative cooling demands are high and nest sites are not near abundant and permanent water sources (Lecomte, Gauthier & Giroux, 2009). This is despite these birds nesting in the Arctic, where water availability is generally not a limiting factor. The lower nesting success is likely due to adults spending more time making drinking trips and leaving nest sites exposed to predation (Lecomte *et al*., 2009).

### (2) Temporal patterns of drinking

Temporal partitioning of drinking is common. Fisher *et al*. (1972) proposed that drinking birds typically fall within one of three temporal drinking categories: (*i*) two relatively short periods typically in the early morning and late afternoon with no drinking during the day; (*ii*) similar patterns to that of the first category but birds may engage in some drinking throughout the day; and (*iii*) drinking throughout the day without any well‐defined temporal peaks. During hot periods, birds may shift drinking to the first category where they are confined to drink during cooler times of the day to avoid intense solar radiation associated with water sources in exposed sites. For instance, Bourke's parrots (*Neopsephotus bourkii*) drink before sunrise or after sunset to avoid the heat of the day (Davies, 1982), a bimodal pattern often observed in parrot species [including Mulga parrots (*Psephotus varius*), Port Lincoln parrots (*Barnardius zonarius*) and galahs (*Eolophus roseicapillus*); Fisher *et al*., 1972] inhabiting hot areas. By contrast, smaller species such as zebra finches (*Taeniopygia guttata*) and Cape sparrows (*Passer melanurus*) are typically considered category 3 drinkers as they continue to drink throughout the day due to their high water requirements (Davies, 1982) but under laboratory conditions, zebra finches have been shown to survive and even breed without access to free water (Prior, Heimovics & Soma, 2013). Obligatory drinkers depend on daily drinking bouts (see Table 2), but these bouts may vary temporally within taxa. For example, only four of 16 sandgrouse species predominantly drink after sundown, while the remaining 12 species typically drink in the morning 2–4 h after sunrise (Ward, 1972). The storage capacity of water also influences water visitation rates, where birds with larger water storage capacity reportedly visit water only once or twice a day, strategically avoiding the hottest part of the day (Fisher *et al*., 1972). These examples allude to the fluidity of drinking classifications, shaped by abiotic and biotic factors, which we elaborate on in Section V.

### (3) Seasonality as a driver of drinking

Whether or not a species drinks may also be driven by seasonal fluctuations in diet, water availability and water demand, influencing the hygric niche (Boyle *et al*., 2020). During hot summers, some species may become more reliant on drinking due to the increased water demand for evaporative cooling purposes. Species may also become constrained in their movement patterns, spending proportionally more time around water sources during the dry season, particularly in already water‐deprived ecosystems (Delgado‐Martínez *et al*., 2023). For example, in the Namib Desert, lark‐like buntings (*Emberiza impetuani*) were consistently found near water sources during the dry season but dispersed once rainfall occurred between January and March. Willoughby & Cade (1967) suggested that the moisture available in green grass shoots, sprouting seeds, and insects during this period likely met the birds' hydration needs, even during the heat of summer and the energetically demanding breeding season. Similar patterns were noted for the North American black‐throated sparrow (*Amphispiza bilineata*; Smyth and Bartholomew, 1996) and southern African frugivorous red‐eyed bulbul (*Pycnonotus nigricans*; Prozesky, 1975). Specifically, in October, when red‐eyed bulbuls were feeding on berries and fruits and the average maximum *T*_air_ reached 26.9 °C, the mean number of individuals drinking each hour was approximately 285. By contrast, on a day in December with a peak *T*_air_ of 37.8 °C, the hourly drinking rate dropped sharply to just 20 birds (Skead, 1975*a*; Abdu *et al*., 2018; Smit *et al*., 2019). Water pressures during dry seasons may also force species to travel long distances in search of water, resulting in distributional shifts as they track seasonal water availability (Tieleman *et al*., 2004). Widespread species occupying diverse habitats may also exhibit intraspecific variations in drinking behaviours. For instance, scaly‐feathered weavers (*Sporopipes squamifrons*) are typically classified as non‐drinkers (Herremans, 1997; Smit *et al*., 2019) but commonly occupy habitats across a rainfall gradient and are often observed and recorded opportunistically drinking in huge numbers, when water is available. Thus, these birds may not be drinking regularly in areas such as the central Kalahari, southern Africa, where water is a limited resource, but may engage in drinking more frequently in areas of semi‐arid savannas north of Pretoria, South Africa, where water is seasonally available (Skead, 1975*a*). However, restricted species can also exhibit considerable intraspecific variation in drinking behaviour. For instance, Willoughby (1971) observed variation in Stark's lark (*Spizocorys starki*) where some individuals within a population would not drink every day, while others routinely visited water sources to drink daily.

### (4) The role of diet on drinking dependence in birds

Building on these spatial and temporal patterns, dietary composition emerges as a key modulator of drinking dependence. Generally, drinking dependence is related to diet and the ability to acquire sufficient water from it (Cade, 1965; Macmillen, 1990). Consequently, functional groups based on dietary requirements are often used to provide insight into patterns of drinking behaviour and the categorisation of species as drinkers *versus* non‐drinkers (Tischler, Dickman & Wardle, 2013; Smit & McKechnie, 2015). For instance, granivorous species, which typically obtain very little water content from dry seeds (~10%), are often more reliant on drinking surface water than insectivorous, frugivorous, and carnivorous bird species whose diets typically provide high quantities of moisture (varies seasonally), ranging from 50% to 90% water content (Table 1) to meet daily hydration needs (Cade, 1965; Prozesky, 1975). However, classifications based on dietary guilds are not fixed and could shift depending on environmental conditions experienced at a given time. For instance, carnivorous species such as tawny eagles (*Aquila rapax*) or bateleurs (*Terathopius ecaudatus*) that have not acquired sufficient water from their diet could, and likely would, drink surface water if available. Moreover, high‐protein diets can lead to increased uric acid formation, decreased abdominal fat and higher water intake, thus increasing water demand (Marks & Pesti, 1984). As such, the exact effects of diet on surface water consumption are complex and likely vary among individuals and taxa, but such ideas have received limited attention. For example, physiological models and empirical studies indicate that high‐protein diets (e.g. 30–78% protein) can elevate uric acid production and excretion, potentially increasing water demand by 20–30% in some taxa to facilitate nitrogenous waste removal (Cade, 1965; Evans, Scholz & Mongin, 1971; Goldstein, Guntle & Flaugher, 2001). In house sparrows (*Passer domesticus*), urate clearance rates were 3.5 times higher on a 30% protein diet compared to 8% protein, while in chicks, rates were 4.3 times higher on 78% *versus* 23% protein diets (Goldstein *et al*., 2001), underscoring the heightened renal water needs that could shift species from opportunistic to regular drinkers along the continuum (Fig. 1; Table 2). Table 1 provides typical water yields from common diets.

Dietary requirements and the associated water derived from the diet also vary spatially and temporally, particularly in response to seasonality and rainfall (Davies, 1982; Tischler *et al*., 2013; Boyle *et al*., 2020). The onset of rainfall drives primary productivity, and the emergence of insects and other invertebrates, which may be water‐rich food sources (Boyle *et al*., 2020), potentially reducing an individual's reliance on surface water, influencing time–activity budgets (e.g. Tieleman & Williams, 2002) and how these species use the thermal landscape (e.g. Orolowitz *et al*., 2023). Additionally, foraging after rainfall, or during periods of high fog, may lead to increased water intake if food sources are wet, particularly in highly hygroscopic seeds (Prozesky, 1975). This increased water intake may be sufficient to meet water requirements, with some granivorous birds abstaining from drinking until the seeds have dried (Prozesky, 1975). Similarly, Skead (1975*a*) observed species such as the green‐winged pytilia (*Pytilia melba*), blue waxbill (*Uraeginthus angolensis*), violet‐eared waxbill (*Uraeginthus granatinus*), and black‐cheeked waxbill (*Estrilda erythronotos*) showing a marked decline in their reliance on free water during the wet season (spring, summer and early autumn), likely because of their intake of water‐rich grass, seeds and insects which provide sufficient moisture for their physiological needs. By contrast, during the dry season, these birds shift to a predominantly dry seed diet and are highly dependent on drinking.

## V. CLASSIFYING SPECIES BASED ON DRINKING HABITS

Given the complexities of drinking behaviour and the role of abiotic and biotic pressures driving these behaviours, our review suggests that the typical dichotomous classification of drinking is likely rather a continuous, flexible classification (Fig. 1, Table 2). The continuum extends from taxa that are heavily reliant on drinking (e.g. sandgrouse, Pterocliformes) to those that obtain all their water through diet and metabolism (e.g. red larks, *Calendulauda burra*, and mistletoebird, *Dicaeum hirundinaceum*). Along this gradient, abiotic (e.g. season, diet) and biotic (e.g. predation, competition) pressures may influence where a species or individual falls along this spectrum within a given environment, at a given time (Fig. 1). For instance, during a heatwave event, southern pied babblers (*Turdoides bicolor*) may shift from opportunistic drinkers to heat‐induced drinkers and back to opportunistic drinkers once temperatures subside (Bourne *et al*., 2021, 2021). Similarly, zebra finches are typically considered regular drinkers, but when water is experimentally removed, these birds can still survive and even breed, despite significant effects on female reproductive physiology and readiness (Prior *et al*., 2013). Given the fundamental role of abiotic and biotic pressures, we propose a revised classification, while emphasising that species may fall into one or more categories depending on their biology and the environmental conditions experienced at a given time.

## VI. EMERGING QUESTIONS AND FUTURE RESEARCH OPPORTUNITIES

Global air temperatures are increasing at an alarming rate, and an improved understanding of the determinants of water acquisition, maintenance and loss is crucial for assessing potential impacts of this warming. Our review sheds light on the complexities associated with understanding water‐intake habits and how abiotic and biotic factors likely influence the classification of avian drinking behaviour. In our attempt to highlight and explore this complex trait, we list in Table 3 several topics, pressing future research questions and potential methodological advances that are necessary if we aim to use drinking as an ecological trait. The emerging questions essentially fall within three broad categories relating to physiology, behaviour and ecology.

**Table 3:** Emerging questions on the drinking dependency of birds in relation to physiology, behaviour and ecology.

	Question level	Question
Physiology	Overarching	Do patterns of physiology differ predictably based on drinking dependence?
	Specific examples	(1) Does organ morphology differ in species with differing drinking classifications? (2) Do patterns of thermoregulation differ predictably depending on drinking dependence across habitat types?
Behaviour	Overarching	How do observations of drinking change drinking classifications?
	Specific examples	(1) Does increasing the number of observations of birds typically classified as non‐drinking increase the likelihood of observing them drinking? (2) Are there specific environmental drivers (e.g. heatwaves) that lead to non‐drinkers needing to drink? (3) Does the removal of water‐rich prey items drive non‐drinking birds to drink? (4) Do sex, age and/or developmental stage influence drinking dependence?
Ecology	Overarching	Does drinking behaviour differ predictably at a spatial, seasonal and/or temporal level?
	Specific examples	(1) How does drinking behaviour differ within a species that inhabits diverse environments with differing water availability? (2) What methods or criteria should be used to classify birds as non‐drinking or drinking?

While the mechanisms of avian water acquisition, retention and loss have been widely studied, far fewer studies have set out to quantify drinking and determine quantitatively how patterns of thermoregulatory traits differ depending on drinking dependence (but see Czenze *et al*., 2020). Do obligate drinkers, for instance, allow for greater EWL than non‐drinkers to maintain *T*_b_ below lethal limits as a result of their high water intake? Continuing within this research theme, do traits associated with thermoregulation (e.g. EWL, *T*_b_) scale predictably across our proposed drinking classifications? Additional potential research avenues to explore involve how anatomical traits and morphology vary depending on drinking dependence.

A common theme that our review highlights is that drinking behaviour is likely a flexible trait, but the extent to which this trait is flexible remains uncertain. To support our understanding of this trait, more observations and direct measurements of when and how birds use water are needed. We urge field biologists to devise and standardise field studies to document drinking and non‐drinking behaviour, and investigate whether increased observations correlate with a greater likelihood of observing non‐drinkers *drink* (Table 3). Similarly, developing behaviour profiles for species using time–activity budgets would allow us to identify novel behaviours and determine whether these are anomalies that do not change their typical drinking classification. Following this line of enquiry, a potentially interesting research question expands on the work done by Tsurim *et al*. (2008) using the experimental manipulation of water and food availability, to include exposure to heat. The inclusion of heat exposure may allow us to determine how the interaction between thermoregulatory demand and water availability influences drinking behaviour, and whether non‐drinking species will indeed drink (given only water‐deprived food options) to accommodate these increased demands under hot conditions. For example, in a pilot study, B. Coulson, M.T. Freeman & A.E. McKechnie (unpublished data) observed that approximately 92% of Afromontane forest bird species, and 74% of individuals, investigated (22 out of 24 species; 125 individuals of 168), willingly drank water following heat exposure during respirometry trials, providing the basis for several emerging questions around water consumption and acute heat exposure.

Another potentially important question relates to understanding and quantifying trade‐offs associated with predator avoidance and water demand, particularly given anticipated rises in *T*_air_. Subsequently, exploring the ecology of drinking at varying spatial and temporal scales is important (Table 3). How, for example, do species that are distributed across diverse habitats varying in water availability differ intraspecifically in their drinking habits? In the context of global change, it may be useful to investigate whether more frequent and longer drinking bouts arising from hot weather are associated with sublethal fitness consequences. Does increased drinking behaviour, for instance, decrease foraging time and thus nutritional status and body condition of adults? In addressing these broad physiology‐, behaviour‐ and ecology‐related questions, we may also gain clarity on the methods and criteria that should be used to group individuals based on drinking dependencies.

The gaps in our understanding of drinking ecology remain vast and provide opportunities for novel and important research. Addressing these gaps will require not only more data but also innovative methodological approaches that make previously intractable questions feasible to answer. Accurately quantifying free water intake remains one of the most persistent methodological challenges in avian ecophysiology. Many species drink from cryptic sources such as dew, tree forks, or rainwater pooled in hollows, making direct observations difficult or near impossible. Even at accessible water points, repeated visits by the same individuals can confound counts of drinking events unless birds are individually marked. Progress will require combining emerging technologies with established physiological and behavioural tools. For instance, integrating stable isotope tracers with field observations could refine water turnover estimates, building on respirometry protocols where stepped (rapid *T*_air_ increments) and steady‐state exposures yield comparable thermoregulatory results (Short, Freeman & McKechnie, 2022). Remote cameras and automated monitoring systems, particularly when integrated with passive integrated transponder (PIT) tags, radio‐frequency identification (RFID) readers, or artificial intelligence (AI)‐based image recognition, can provide fine‐scale data on the timing and frequency of visits by known individuals (Pacheco‐Fuentes *et al*., 2025). Global positioning system (GPS)‐loggers with accelerometers can reveal drinking behaviour away from monitored points (collection of reliable data sets using this technique currently is restricted to large birds), while stable isotope tracers and doubly labelled water approaches remain indispensable for quantifying water turnover in free‐living birds (Smit *et al*., 2019). Advances in environmental DNA sampling from water sources also offers exciting prospects for detecting which species use these water sources (Holman *et al*., 2019; Flitcroft *et al*., 2025). Experimental manipulations, such as provision or exclusion of water points, offer another route to identifying thresholds for drinking behaviour. Although field studies of drinking behaviour remain limited, recent work has begun to quantify individual drinking patterns in free‐living birds (Pacheco‐Fuentes *et al*., 2025). Complementing these field‐based approaches, laboratory studies using captive colonies also provide valuable insights into drinking ecology, where controlled dehydration experiments can reveal physiological thresholds and inform inferences about species' reliance on water in natural conditions (Prior *et al*., 2013). Together, these approaches outline a starting point for closing existing knowledge gaps around avian water balance and drinking habits. However, while these approaches hold great promise, researchers should remain vigilant of inherent biases, such as the over‐representation of arid‐zone species data in camera trap studies, which may skew insights towards water‐scarce environments, and prioritise multi‐habitat sampling to ensure more representative and generalisable findings.

## VII. CONCLUSIONS

(1) Water is a fundamental currency of life, but the mechanisms that underlie water acquisition, retention, and loss in avifauna are complex.
(2) Our review highlights that a species' ecology, physiology, behaviour and surrounding environment drive water dependencies and that the dichotomous classification of drinking *versus* non‐drinking may not be ecologically relevant.
(3) Understanding drinking as a trait may provide crucial insights into the mechanisms driving species vulnerabilities to changing climates, but caution is needed when including drinking as a trait in climate vulnerability assessments. Closing these gaps will depend on deploying innovative methodological approaches to quantify drinking accurately, thereby advancing our ability to link water balance, behaviour, and fitness in free‐living birds. This is particularly urgent given emerging threats, such as rising temperatures and humidity levels, that exacerbate hyperthermia risks by constraining evaporative cooling efficiency [e.g. as demonstrated in recent studies on tropical and arid avifauna (Conradie *et al*., 2020; Freeman *et al*., 2024; Coulson *et al*., 2025)], which could disproportionately impact species at the drinking‐dependent end of the continuum and amplify mass‐mortality events in a warming world.

## AUTHOR CONTRIBUTIONS

S.R.C. and M.T.F. contributed equally to the conceptualisation, writing and editing of this review.

## CONFLICT OF INTEREST STATEMENT

None of the authors have a conflict of interest to disclose.

## Data Availability

Data sharing not applicable to this article as no datasets were generated or analysed during the current study.
